# A flexible codon in genomically recoded *Escherichia coli* permits programmable protein phosphorylation

**DOI:** 10.1038/ncomms9130

**Published:** 2015-09-09

**Authors:** Natasha L. Pirman, Karl W. Barber, Hans R. Aerni, Natalie J. Ma, Adrian D. Haimovich, Svetlana Rogulina, Farren J. Isaacs, Jesse Rinehart

**Affiliations:** 1 Department of Cellular & Molecular Physiology, Yale University, New Haven, Connecticut 06520-8114, USA; 2 Department of Molecular, Cellular and Developmental Biology, Yale University, New Haven, Connecticut 06520-8114, USA; 3 Systems Biology Institute, Yale University, New Haven, Connecticut 06520-8114, USA

## Abstract

Biochemical investigation of protein phosphorylation events is limited by inefficient production of the phosphorylated and non-phosphorylated forms of full-length proteins. Here using a genomically recoded strain of *E. coli* with a flexible UAG codon we produce site-specific serine- or phosphoserine-containing proteins, with purities approaching 90%, from a single recombinant DNA. Specifically, we synthesize human MEK1 kinase with two serines or two phosphoserines, from one DNA template, and demonstrate programmable kinase activity. Programmable protein phosphorylation is poised to help reveal the structural and functional information encoded in the phosphoproteome.

## Abstract (web summary)

The effects of protein phosphorylation, a common post-translational modification, are difficult to study using recombinant proteins. Here the authors use genomically engineered *E. coli* to enhance translation systems that express phosphor-serine containing proteins, and use these systems to produce phosphorylated MEK1 kinase.

## Graphical abstract

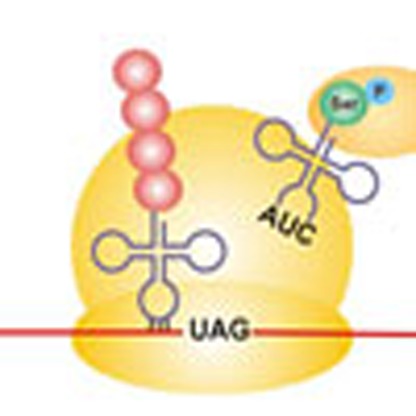

Protein phosphorylation modulates most cellular functions and is controlled by networks of kinases and phosphatases that add or remove phosphate groups at precisely defined positions. Large-scale phosphoproteomics efforts have mapped phosphorylation sites on the majority of human proteins^1^,^2^. Our knowledge of the phosphoproteome has outpaced our understanding of which protein kinases phosphorylate these important sites. Nature has evolved elaborate mechanisms to ensure proper kinase/substrate pairings^3^. Recombinant phosphoprotein synthesis is impeded by the fact that most of these pairs are unknown. We recently described a solution to this problem using *Escherichia coli* strains engineered to incorporate genetically encoded phosphoserine (Sep) into recombinant proteins by employing an orthogonal translation system (SepOTS)^4^. The SepOTS uses a Sep aminoacyl-tRNA synthetase (SepRS) to charge Sep onto a UAG-decoding tRNA^Sep^ and an engineered elongation factor Tu (EF-Sep) that delivers Sep–tRNA^Sep^ to the ribosome^4^. The SepOTS can direct phosphoserine incorporation into physiologically relevant positions within proteins without any knowledge of upstream kinases and thus provides a simplified platform for phosphoprotein synthesis.

The first described iteration of the SepOTS consisted of two plasmids housing genes for SepRS, EF-Sep and one gene copy of tRNA^Sep^ (here referred to as SepOTSα)^4^. SepOTSα was functional in a standard BL21 *E. coli* strain, but inefficiently encoded Sep at UAG amber codons due to competition with release factor one (RF1). This barrier has long been recognized in the field of non-standard amino-acid (NSAA) incorporation and has compromised the yield and purity of NSAA-containing proteins. We recently demonstrated that recoding^5^ the native TAG sites terminating seven essential genes in the *E. coli* genome to TAA enabled RF1 deletion and enhanced Sep incorporation^6^. This partially recoded cell (EcAR7.ΔA) enhanced UAG read-through, but exhibited severe growth impairment and suppression with natural amino acids. The deletion of RF1 causes ribosome stalling on the non-recoded, UAG-containing mRNAs, and protein instability from proteome-wide UAG read-through is elevated in the presence of the SepOTS^6^,^7^ (Fig. 1a). We found that Sep incorporation at UAG sites was enhanced in EcAR7.ΔA, but we quantified natural amino-acid incorporation accounting for as much as 60% of UAG decoding^7^, indicating that further advances in strain and SepOTS engineering were required. We recently introduced the first genomically recoded organism (C321.ΔA) where all 321 TAG sites in the genome were reassigned to TAA^8^. This strain not only tolerated the deletion of RF1 but also eliminated all RF1-knockout-associated growth defects observed in partially recoded strains. Furthermore, C321.ΔA exhibited muted UAG read-through by natural aminoacyl-tRNAs that compete with NSAA incorporation via OTSs^8^ (Fig. 1a). The properties of C321.ΔA suggested that this strain would provide the ideal setting for Sep incorporation, offering a completely open and assignable UAG codon in a background that does not suffer from the low purity and compromised fitness observed in other RF1-knockout strains^6^,^7^.

Here we demonstrate the utility of genomically recoded *E. coli* to produce phosphoproteins via site-specific incorporation of Sep with an enhanced version of the SepOTS. We take advantage of the flexible UAG codon to genetically program recombinant human MEK1 kinase activity, and we characterize our platform based on phosphoprotein yield, purity and positional bias. Overall, we conclude that our expression system enables robust expression of diverse phosphoproteins for potential biochemical and phosphoproteomic applications.

## Results

### Recoding *E. coli* permits increased phosphoprotein purity

**Figure 1:**
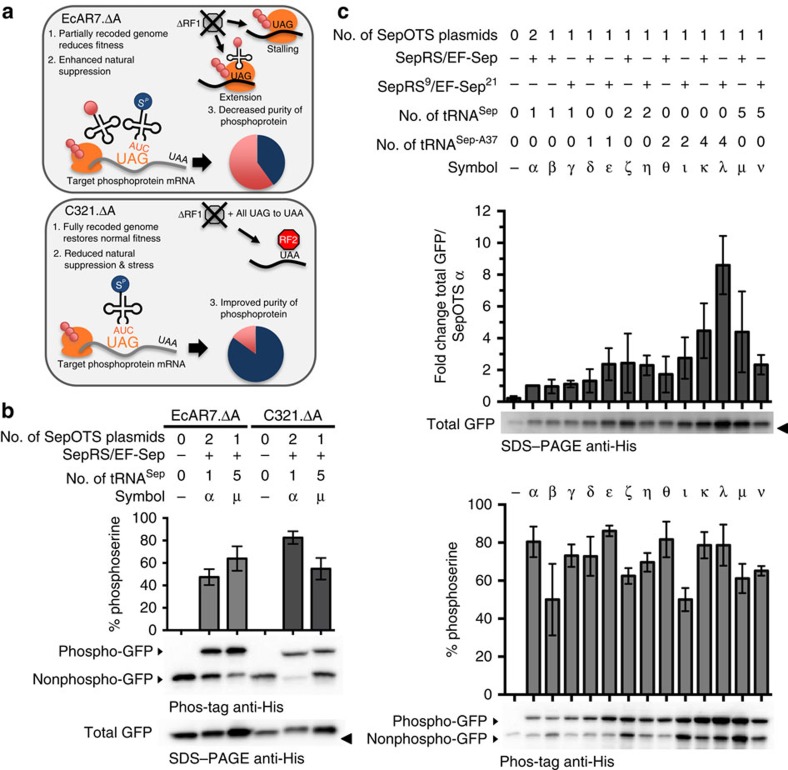
Development of SepOTS variants for improved phosphoprotein yields and purity in C321.ΔA. (**a**) Schematic of Sep incorporation into a target recombinant phosphoprotein in the setting of the partially recoded EcAR7.ΔA and the fully recoded C321.ΔA strains. The 314 TAG-containing non-recoded loci in EcAR7.ΔA lead to ribosome stalling and increased near-cognate suppression. Sep incorporation at UAG codons is reduced due to competition for the target UAG by near-cognate suppression. The fully recoded C321.ΔA cell restores natural protein synthesis by restoring release factor function at all 321 recoded loci. Improved cellular fitness and reduced near-cognate suppression significantly improve the purity of recombinant phosphoproteins. (**b**) Phospho-GFP expression using two SepOTS variants (two-plasmid system including SepRS, EF-Sep and 1 × tRNA^Sep^ denoted SepOTSα and one-plasmid system including SepRS, EF-Sep and 5 × tRNA^Sep^ denoted SepOTSμ) in the EcAR7.ΔA versus C321.ΔA strains. A UAG codon at position 17 directs Sep incorporation into GFP. Purity was determined by Phos-tag gel shift and western blot and comparing intensities of the upper band (phospho-GFP) and the lower band (non-phospho-GFP). Error bars report s.d. from six biological replicates. Normalized loading was approximated by regular SDS–PAGE analysis of total GFP expression. Extended data is shown in Supplementary Fig. 1. (**c**) Phospho-GFP (Sep at position 17) expression using 13 SepOTS variants in the C321.ΔA strain. Fold change reflects GFP expression for each SepOTS variant compared with SepOTSα. Loading was normalized by OD_600_ measurement. Error bars report s.d. of three biological replicates. Purity of phospho-GFP was determined by Phos-tag gel shift and western blot. Error bars report s.d. from three biological replicates. Blots in (**b**) and (**c**) were performed using antibody against C-terminal 6xHis tag in GFP. The solid triangles denote the 25-kDa molecular weight marker. Phos-tag gels are run without molecular weight standards.

We aimed to leverage the enhanced properties of C321.ΔA to further improve the SepOTS and to generate recombinant phosphoproteins with higher yield and purity. To evaluate SepOTS variants, we utilized an established green fluorescent protein (GFP) reporter in which position 17 is permissive to a glutamate-to-Sep substitution^6^. We recently used this reporter and quantitative mass spectrometry to show that using five tRNA^Sep^ gene copies in our SepOTS plasmid (here within referred to as SepOTSμ) increases the yield and purity of phosphoproteins produced in EcAR7.ΔA^7^. Our analysis showed that Sep incorporation reached only 40% and UAG read-through by native aminoacyl-tRNAs accounted for 60% of the UAG decoding^7^. To efficiently compare and track this phenomenon across different strains, we adopted a Phos-tag gel shift assay^9^ in which phosphoserine-containing proteins are separated from non-phosphorylated proteins and can be quantified on the same western blot^10^,^11^,^12^. The gel shift assays showed that increasing tRNA^Sep^ gene copy number from one to five raised Sep incorporation at one UAG in EcAR7.ΔA from 40 to 60% and was generally in good agreement with our previous studies^7^ (Fig. 1b, Supplementary Fig. 1). As a first step, we moved these two SepOTS variants into C321.ΔA. SepOTSα showed increased Sep incorporation to over 80% in C321.ΔA compared with only 40% in EcAR7.ΔA (Fig. 1b). This was the first clear indication that the C321.ΔA background significantly enhanced the ability of SepOTSα to encode Sep at higher purity. Interestingly, there was little difference in phospho-GFP purity using the SepOTSμ with 5 tRNA^Sep^ gene copies and suggested that simple tRNA gene copy number manipulation would not be sufficient to further enhance SepOTS performance in C321.ΔA.

### Modifying SepOTS to enhance phosphoserine incorporation

The properties of C321.ΔA, in particular the increased fitness and reduction of strong UAG read-through observed in partially recoded strains^7^,^8^ that directly competes with Sep insertion, inspired us to perform a more extensive assessment of SepOTS variants in the C321.ΔA background. To accomplish this, we assembled combinations of known and novel SepOTS components and compared the total phospho-GFP synthesis and purity in C321.ΔA. We used the enhanced SepRS9 and EF-Sep21 variants and multiple gene copies of tRNA^Sep^ known separately to increase SepOTS performance^7^,^13^. We also introduced a new tRNA^Sep^ variant containing a G37A substitution (tRNA^Sep-A37^) known to improve UAG read-through in other natural suppressor tRNAs^14^. We hypothesized that this tRNA^Sep^ mutation would enhance Sep decoding by stabilizing anticodon base stacking in the ribosome^15^. We compared relative phospho-GFP expression for 12 different SepOTS combinations (renamed SepOTSβ–SepOTSν for simplicity) to the original two-plasmid system SepOTSα (ref. 4) (Fig. 1c). The most dramatic increase was observed using SepOTSλ, which contained enhanced SepRS9, EF-Sep21 and four gene copies of tRNA^Sep-A37^ and yielded approximately ninefold elevation in GFP expression compared with SepOTSα. However, the variants containing SepRS9 and EF-Sep21 did not always outperform the original SepOTSα in the combinations tested. We did not observe a consistent correlation between increased tRNA gene copy number and increased phospho-GFP production, but observed that four gene copies of tRNA^Sep-A37^ performed better than five gene copies of tRNA^Sep^. This suggests that the G37A mutation increased the performance of the tRNA^Sep^ synergistically with a tRNA gene copy number effect. Northern blot analysis of these cells confirmed that tRNA^Sep^ levels generally correlate with gene copy number for all SepOTS variants (Supplementary Fig. 2). Although we cannot fully explain the fluctuations in the expression patterns, we speculate that perhaps tRNA gene copy number may alter tRNA base modifications and contribute to the patterns observed. When we examined the purity of phospho-GFP with gel shift we observed that most of the SepOTS variants produce consistently higher phosphoprotein purity in C321.ΔA than the variants tested in the partially recoded EcAR7.ΔA (Fig. 1b,c). We noted that the increased protein yield from twofold with SepOTSε to ninefold with SepOTSλ did not compromise the purity of Sep insertion at UAG since both OTS systems achieved purities between 80 and 90% in the C321.ΔA strain (Fig. 1c).

### Flexible codon for incorporation of serine or phosphoserine

The flexibility of amber codon amino-acid assignment in C321.ΔA presents the possibility of synthesizing proteins encoding either Ser or Sep at desired positions within the recombinant protein (Supplementary Fig. 3a). Taking advantage of this synthetic flexibility, we employed a known amber suppressor tRNA SupD to incorporate Ser at UAG codons^16^. We envision this modularity having important practical applications for the incorporation of either Sep or Ser at amber codons in large synthetic gene libraries, obviating the need for separate DNA pools encoding Sep and Ser. To test amber codon flexibility in an enzyme, we used beta-lactamase (Bla) since its catalytic site requires Ser at position 68 to produce ampicillin-resistant cells^17^. A Bla variant replacing S68 with a TAG codon (S68TAG Bla) was then created. C321.ΔA cells transformed with SupD and S68TAG Bla plasmids were resistant to ampicillin and confirmed that the amber codon could be used to encode serine (Supplementary Fig. 3b). C321.ΔA transformed with SepOTSλ and S68TAG Bla plasmids to encode Sep at the amber codon yielded expression of inactive Bla and no cell growth in the presence of ampicillin (Supplementary Fig. 3b). This experiment also validates the stability of Sep incorporation at position 68 in the Bla protein, since robust *in vivo* Sep dephosphorylation to Ser would have produced an active Bla enzyme. These results show that the amber codon in C321.ΔA can be used to create an active and inactive enzyme from the same gene. Furthermore, this demonstrates that the phosphorylation state and function of a protein can be programmed by simply employing different translation machinery for the amber codon.

### Programmable active kinase production

We next wanted to use our enhanced SepOTS systems and the flexible UAG to control phosphoserine-related function in a physiologically relevant mammalian protein containing more than one phosphoserine. Mitogen-activated protein kinase kinase 1 (MEK1) directly controls a broad range of cell cycle functions, is implicated in oncogenesis^18^, and like many kinases^19^,^20^,^21^,^22^ is widely believed to require phosphorylation at two sites for activity. To measure the robustness of our experimental systems we synthesized MEK1 with genetically encoded Sep at positions 218 and 222 (MEK1-S^P^S^P^) in its activation segment. We first used SepOTSμ (refs 7, 23) to compare MEK1-S^P^S^P^ expression levels in BL21 RF1^+^, EcAR7.ΔA and C321.ΔA. MEK1-S^P^S^P^ expression in BL21 RF1^+^ was undetectable due to RF1 activity, whereas both EcAR7.ΔA and C321.ΔA RF1-deficient backgrounds easily produced detectible levels of MEK1-S^P^S^P^ (Supplementary Fig. 4). C321.ΔA has a growth curve with a steep log phase and final OD_600_ closer to wild-type cells^8^ and consistently outperforms the poor growth characteristics of EcAR7.ΔA^6^ (Supplementary Fig. 5). This substantial boost in fitness, and the enhanced SepOTS performance in the C321.ΔA strain, rendered it the optimal strain for MEK1-S^P^S^P^ expression.

**Figure 2:**
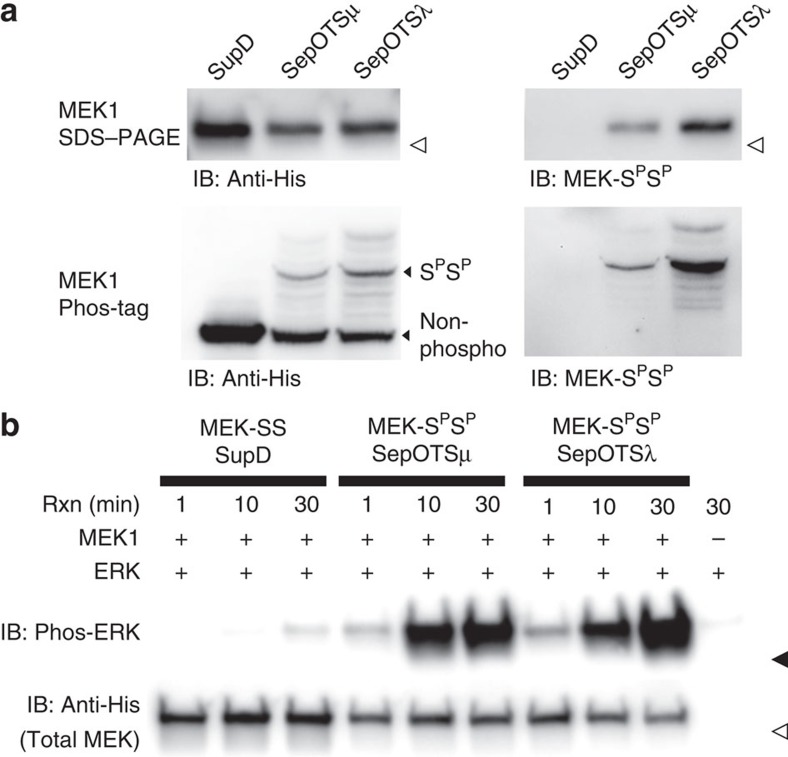
A single-gene system for robust production of phosphorylated and non-phosphorylated human kinases. (**a**) Analysis of MBP-MEK1 S218TAG/S222TAG expression in the fully recoded C321.ΔA strain using plasmids that enable incorporation of Ser (one-plasmid system encoding SepRS, EF-Sep and 2 × tRNA^SupD^ denoted SupD) or Sep (one-plasmid system encoding SepRS, EF-Sep and either 5 × tRNA^Sep^ denoted SepOTSμ or 4 × tRNA^Sep-A37^ denoted SepOTSλ) at amber codons. Total expression was evaluated by western blot using an antibody against the C-terminal 6xHis tag on MBP-MEK1. Loading was normalized by OD_600_ measurement. Presence of phosphorylated MBP-MEK1 was verified using a phosphospecific antibody (extended data shown in Supplementary Fig. 6a). Phosphoprotein purity was determined by Phos-tag gel shift and western blot by comparing the relative intensity of the upper bands compared with the lower band, corresponding to the phosphorylated and non-phosphorylated forms of MBP-MEK1, respectively. (**b**) Assay of MBP-MEK1 kinase activity monitored by phosphorylation of a kinase-dead ERK2 substrate *in vitro*. Representative blots showing phosphorylated (anti-Phos-ERK) and total MEK (anti-His) are shown. Assays and westerns were performed in triplicate and representative results are shown. The open and solid triangles denote 75- and 37-kDa molecular weight markers, respectively. Phos-tag gels are run without molecular weight standards.

To further investigate MEK1 synthesis we used the highest performing SepOTS variant, SepOTSλ, and found expression of MEK1-S^P^S^P^ was twofold higher (∼2 mg per l culture) than with SepOTSμ (∼1 mg per l culture, Fig. 2a). In parallel, we used SupD in C321.ΔA with the same *MEK1* gene (TAGs at 218 and 222) to produce inactive MEK1 containing Ser at positions 218 and 222 (Fig. 2a). MEK1-S^P^S^P^ production was confirmed with a commercially available phosphospecific antibody for positions 218 and 222 (Fig. 2a) and further characterized by examining singly phosphorylated forms of MEK1 (MEK1-S^P^S and MEK1-SS^P^) by Phos-tag gel shift (Supplementary Fig. 6a). These studies confirmed the synthesis of singly and doubly phosphorylated MEK1 but revealed that the MEK1 phosphospecific antibody could readily detect both MEK1-S^P^S^P^ and MEK1-SS^P^, but not MEK1-S^P^S. We also analysed the MEK1-SS, SS^P^ and S^P^S^P^ variants by mass spectrometry and verified Sep incorporation (Supplementary Fig. 6b and Supplementary Table 1). Despite our best efforts, we were unable to directly observe the doubly phosphorylated peptide likely due to poor ionization. To overcome this problem, we treated MEK1-S^P^S^P^ with calf intestinal alkaline phosphatase to dephosphorylate the protein. Calf intestinal alkaline phosphatase-treated MEK1-S^P^S^P^ produced a peptide containing two serine residues at the S218 and S222 positions (absent in untreated MEK1-S^P^S^P^ sample), confirming that Sep was originally inserted at both positions within the protein. We next used our established data analysis techniques to identify natural amino acids incorporated at the two different UAG sites in the MEK1 activation loop^7^ (Supplementary Table 1). These non-phosphorylated MEK1 peptides explain a portion of the unshifted bands detected in our Phos-tag analysis and, consistent with our previous studies^7^,^8^, demonstrate that near-cognate suppression of the amber codon can lead to natural amino-acid incorporations that interfere with SepOTS activity and phosphoprotein purity.

A side-by-side comparison of MEK1 production showed that SepOTSμ and SepOTSλ yielded ∼12% and ∼30% phosphorylated MEK1-S^P^S^P^, respectively (Fig. 2a). While SepOTSλ increased the yield of MEK1-S^P^S^P^ as predicted by the GFP reporter studies, the yield of phosphorylated MEK1 was lower than phospho-GFP. This could be due to instability of phosphorylated MEK or context-specific effects of UAG suppression in the MEK activation segment. To examine protein stability we conducted time course expression studies that showed higher MEK1-S^P^S^P^ phosphoprotein yield with shorter expression times (Supplementary Fig. 6c). We assessed positional effects of Sep incorporation or stability using several singly and doubly phosphorylated forms of GFP (Supplementary Fig. 6d). The differences in phosphoserine incorporation between the GFP variants led us to conclude that UAG context and phosphoprotein stability are both contributing factors to the overall purity of recombinant phosphoproteins.

To demonstrate the utility of the flexible UAG codon in synthesizing active mammalian kinases, we carried out MEK1 *in vitro* phosphorylation assays. MEK1-S^P^S^P^ produced robust *in vitro* ERK2 phosphorylation while non-phosphorylated MEK1 (produced with SupD in C321.ΔA) was completely inactive (Fig. 2b). This demonstrates that the activity of a protein kinase can be programmed by simply using different translational machinery for the UAG codon in C321.ΔA cells. We envision that this flexibility could be leveraged in experiments with large panels or arrays of recombinant enzymes designed to explore the phosphoproteome at greater depth.

## Discussion

These results clearly demonstrate that the physiological properties of protein phosphorylation can be controlled by a programmable genetic code. The flexible UAG codon in C321.ΔA can be manipulated with different OTS systems to produce proteins encoding either Sep or Ser at amber codons. We show that C321.ΔA is an optimal host for phosphoprotein expression with SepOTSε and SepOTSλ spanning a ninefold expression range without compromising purity. These systems provide a platform to reveal the functional consequences of serine phosphorylation. As a proof of principle, we reproduced the phosphorylation events underlying the MEK1 activity switch that controls a human signalling cascade. Our programmable UAG can be used to model other important kinase regulatory switches to enable applications such as kinase substrate discovery and screens for novel kinase inhibitors. More broadly, different pairs of natural suppressor tRNAs and OTSs could be introduced into C321.ΔA to explore more types of post-translational modification^24^. Certainly, exploring the function of protein phosphorylation in more enzymatic classes and across different species should now be possible. We recently showed that our C321.ΔA-based phosphoserine systems can be used for *in vitro* protein synthesis platforms, which may further extend the experimental landscape^25^. Guided by the large number of physiological protein phosphorylation sites^2^, systematic investigations into the structure and function of any phosphoproteome could be within reach.

## Methods

### Transformations and strain storage

All *E. coli* strains used in this study were made chemically competent using a standard RbCl_2_ method. EcAR7.ΔA was co-transformed with E17TAG GFP in the modified pCR-Blunt II-TOPO vector with the signified SepOTS version. C321.ΔA was co-transformed with PCRT7 NT Topo tetR pLtetO plasmid containing S2TAG, E17TAG, Q157TAG, S2TAG/E17TAG, E17TAG/Q157TAG GFP, maltose binding protein-MEK1 (MBP-MEK1) (S218TAG/S222), MBP-MEK1 (S218/S222TAG) or MBP-MEK1 (S218TAG/S222TAG) cassettes with the designated SepOTS. BL21 RF1+ was co-transformed with MBP-MEK1 and the signified SepOTS. All strains were stored as frozen glycerol stocks and restreaked on selective Luria–Bertani (LB) agar plates before protein expression. Strains harbouring the following plasmids were grown with the indicated antibiotic concentrations: E17TAG GFP in the modified pCR-Blunt II-TOPO plasmids, 25 μg per ml Zeocin (Zeo); all pCRT7 NT Topo tetR pLtetO plasmids, 100 μg per ml ampicillin (Amp); SupD and all SepOTS plasmids, 25 μg per ml kanamycin (Kan) with the exception of the two plasmid SepOTSα which requires 6 μg per ml tetracycline (Tet) and 25 μg per ml Kan; β-lactamase S68TAG plasmid, 10 μg per ml chloramphenicol (Cam) or 100 μg per ml Amp, as indicated. All cultures were started from a freshly streaked glycerol stock on LB agar plates with the appropriate combination of antibiotics and 0.08% glucose. Detailed information about strain and plasmid generation can be found in the Supplementary Methods. All primer sequences can be found in Supplementary Table 2. Sequences of tRNA-related synthesized genes can be found in Supplementary Table 3.

### GFP variant protein expression

*E. coli* strains were streaked from −80 °C frozen glycerol stocks on LB agar selective plates and grown ∼20 (BL21-based strains), ∼24 (C321.ΔA-based strains), or ∼48 h (EcAR7.ΔA-based strains) at 30 °C. Five colonies were inoculated in 5 ml of LB media supplemented with appropriate antibiotic and 0.08% glucose. Precultures were grown ∼16 h at 30 °C shaking at 230 r.p.m. Cells were diluted to OD_600_ of 0.15 a.u. in 20 ml of LB with antibiotics, 0.08% glucose, 2 mM *O*-phospho-L-serine (Sep), and protein expression was induced with 1 mM isopropyl β-D-1-thiogalactopyranoside and 100 ng per ml anhydrotetracycline. Protein was expressed for 20 h at 30 °C shaking at 230 r.p.m. unless otherwise noted. An equivalent number of cells as 1 ml of OD_600_ 2.5 a.u. was collected and spun down at 4,000*g* for 5 min at 4 °C. Media was aspirated and dry cell pellets were stored at −80 °C. Transformation and retention of the correct SepOTS variant (including tRNA gene copy number) were confirmed by PCR from frozen cell pellets. Cells were resuspended in 40 μl bacterial lysis buffer (50 mM Tris/HCl (pH 7.4), 150 mM NaCl, 1 mM dithiothreitol (DTT), 50 mM NaF, 1 mM NaVO_4_ and 5% glycerol) supplemented with cOmplete mini-EDTA-Free protease inhibitor cocktail tablets (Roche) and 1 × BugBuster detergent (Novagen), and kept on ice for 10 min. Samples were then spun down at 21,000*g* for 7 min at 4 °C and the supernatant (lysate) was transferred to a new tube and stored at −80 °C.

For western analysis, equal volumes of each sample were diluted in 1 × Laemmli buffer (Bio-Rad). Samples were run on a 15-well 4–15% acrylamide gels (Bio-Rad) or on a handcast 15-well 12% acrylamide gel containing 100 μM Phos-tag Acrylamide (Waco Pure Chemical Industries, Inc., AAL-107). Transferred polyvinylidene difluoride (PVDF) membranes were blotted with 1:2,500 Anti-His (6xHis Epitope TAG, PA1-983B, Thermo Fisher Scientific), followed by 1:10,000 DAR–HRP (Peroxidase-conjugated AffiniPure Donkey Anti-Rabbit IgG, 711-035-152, Jackson ImmunoResearch). Signal was detected by enhanced chemiluminescence (Bio-Rad) imaged on a ChemiDoc XRS+CCD camera (Bio-Rad). Densitometry was performed using the Bio-Rad Image Lab software. Three biological replicates (starting from glycerol stocks) were performed for both the total expression and phospho-shifted westerns. Uncropped western blot images corresponding to text and Supplementary Figures are included in Supplementary Figs 7–12.

### MBP-MEK1 recombinant protein expression and purification

The 5-ml precultures were inoculated with 5–20 colonies and grown overnight to confluency in LB media containing 0.08% glucose and antibiotics. The precultures were diluted to OD_600_ 0.15 a.u. in 100 ml LB media containing 0.08% glucose, antibiotics and 2 mM Sep, and were incubated at 30 °C, 230 r.p.m. for ∼3 h to an OD_600_ of 0.8 a.u. Protein expression was then induced with 100 ng per ml anhydrotetracycline and 1 mM isopropyl β-D-1-thiogalactopyranoside, and cultures were grown at 20 °C, 230 r.p.m. for ∼20–22 h unless otherwise noted. An equivalent number of cells as 1 ml of OD_600_ 2.5 a.u. was collected and spun down at 4,000*g* for 5 min at 4 °C. The remaining cells were collected at 4,000*g*, 20 min, 4 °C. Pellets were resuspended in ∼30 ml of the used LB media and transferred to 50-ml centrifuge tubes and centrifuged under the same conditions again. All media was decanted, and pellets were stored at −80 °C.

The 1-ml OD_600_ 2.5 a.u. cell pellets were resuspended in 40 μl bacterial lysis buffer (with protease inhibitors and detergent, as for E17TAG GFP expression above) and kept on ice for 10 min. Samples were then spun down at 21,000*g* for 7 min at 4 °C and the supernatant (lysate) was transferred to a new tube and stored at −80 °C.

For western analysis, equal volumes of OD_600_ normalized lysates were diluted in 1 × Laemmli buffer (Bio-Rad). Samples were run on a 15-well 4–15% acrylamide gels (Bio-Rad) or on a handcast 10-well 7.5% acrylamide gel containing 25 μM Phos-tag Acrylamide. Transferred PVDF membranes were blotted with either 1:2,500 anti-His (6xHis Epitope TAG, PA1-983B, Thermo Fisher Scientific) for total MEK1 or 1:1,000 Anti-MEK-S^P^S^P^ (Phospho-MEK1/2 (Ser217/221), 9154, Cell Signaling Technology), followed by 1:10,000 DAR–HRP. Signal was detected by enhanced chemiluminescence (Bio-Rad) imaged on a ChemiDoc XRS+CCD camera. Densitometry was performed using the Bio-Rad Image Lab software.

Samples for MBP-MEK1 (S218TAG/S222TAG) in C321.ΔA harbouring either the SepOTSλ or SupD plasmids expression time course were expressed as described above, with exception that after induction 1-ml aliquots were removed at denoted time points and spun down at 4,000*g* for 5 min at 4 °C. All media was decanted, and pellets were stored at −80 °C. For western analysis samples were lysed in 80 μl of 2 × Laemmli buffer and ran on a handcast 15-well 7.5% acrylamide gel containing 25 μM Phos-tag Acrylamide. Membranes were blotted with Anti-His as described above.

Cell pellets for purification were thawed in a 37 °C water bath for∼20 s and then put on ice. The pellets were resuspended in 5 ml of bacterial lysis buffer for sonication (50 mM Tris/HCl (pH 7.4), 500 mM NaCl, 0.5 mM EDTA, 0.5 mM EGTA, 1 mM DTT, 1 mg per ml lysozyme, 50 mM NaF, 1 mM NaVO4, 10% glycerol and complete mini-EDTA-Free protease inhibitor cocktail tablets) and incubated on ice for 30 min, followed by sonication. The lysates were centrifuged at 22,000*g*, 15 min, 4 °C and the clarified lysate was transferred to a 15-ml tube, and centrifuged under the same conditions again to remove all remaining insoluble material. The 200 μl bed volumes of Ni-NTA agarose resin (Qiagen Valencia, CA) were transferred to Pierce spin columns (Thermo Scientific Waltham, MA) and resin was equilibrated with 5 ml of Ni-NTA equilibration buffer (50 mM Tris/HCl (pH 7.4), 500 mM NaCl, 0.5 mM EDTA, 0.5 mM EGTA, 1 mM DTT, 50 mM NaF, 1 mM NaVO_4_ and 10% glycerol). The clarified lysate was loaded onto the column via syringe then the column was washed with 10 ml of Ni-NTA wash buffer (50 mM Tris/HCl (pH 7.4), 500 mM NaCl, 0.5 mM EDTA, 0.5 mM EGTA, 1 mM DTT, 50 mM NaF, 1 mM NaVO_4_, 10% glycerol and 20 mM imidazole). Protein was eluted using Ni-NTA elution buffer (50 mM Tris/HCl (pH 7.4), 500 mM NaCl, 0.5 mM EDTA, 0.5 mM EGTA, 1 mM DTT, 50 mM NaF, 1 mM NaVO_4_, 10% glycerol and 250 mM imidazole) and 400 μl elutions were collected in a clean Eppendorf tubes. Each fraction was assessed by SDS–polyacrylamide gel electrophoresis (SDS–PAGE).

The eluates were pooled, concentrated and buffer exchanged into the protein storage buffer (50 mM Tris/HCl (pH 7.4), 150 mM NaCl, 1 mM DTT and 20% glycerol) using a 0.5-ml Amicon Ultra centrifugal filter (Millipore Billerica, MA) and the protein was stored at −20 °C. The protein concentration was estimated by UV280 and by comparing known quantities of BSA standards on an SDS–PAGE gel.

K54R ERK2 was expressed and purified as described for MEK1 above.

### MEK1-S^P^S^P^ kinase activity assay

Kinase activity of purified MBP-MEK1 (S218/S222 or S^P^218/S^P^222) was evaluated by measuring ERK2 phosphorylation. In all, 1.0-μM MBP-MEK1 variants were pre-incubated in kinase activity buffer (50 mM Tris/HCl (pH 7.4), 150 mM NaCl, 1 mM DTT, 20% glycerol, 10 mM MgCl_2_ and 1 mM ATP) at 30 **°**C for 5 min, then 2.5 μM ERK2 substrate was added to the reaction and further incubated at 30 **°**C. A 7.5 μl volume of the reaction was removed at 1, 10 and 30 min time points and quenched with 7.5 μl of 2 × Laemmli sample buffer (Bio-Rad), then heated to 55 °C for 5 min. A negative control was run with only ERK2 substrate for 30 min and quenched in the same manner as kinase-containing samples. The quenched reactions were run on 15-well 4–15% acrylamide SDS–PAGE gels and transferred to a PVDF membrane. Each membrane was cut between the 50–75-kDa protein markers and the bottom portion of the membrane was blotted with 1:1,000 anti-Phos-Erk antibody (Phospho p44/42 MAPK (Erk1/2) (Thr 202/Tyr204), 9101, Cell Signaling Technology) and the top portion of the membrane was blotted with 1:2,500 Anti-His antibody (6xHis Epitope TAG, PA1-983B, Thermo Fisher Scientific) for total MBP-MEK1, followed by 1:10,000 DAR–HRP. Signal was detected by enhanced chemiluminescence (Bio-rad) imaged on a ChemiDoc XRS+CCD camera. The kinase activity assay was run in triplicate using the same purified preparation of MBP-MEK1 kinase and K54R ERK2.

## Additional information

**How to cite this article:** Pirman, N. L. *et al.* A flexible codon in genomically recoded *Escherichia coli* permits programmable protein phosphorylation. *Nat. Commun.* 6:8130 doi: 10.1038/ncomms9130 (2015).

## Supplementary Material

Supplementary Information Supplementary Figures 1-12, Supplementary Tables 1-3, Supplementary Methods and Supplementary References
