# Supplementary material for: A flexible codon in genomically recoded Escherichia coli permits programmable protein phosphorylation
Source: Nat Commun. 2015 Sep 9;6:8130. doi: 10.1038/ncomms9130 (PMC4566969; doi:10.1038/ncomms9130)
Supplement: Supplementary Information — Supplementary Figures 1-12, Supplementary Tables 1-3, Supplementary Methods and Supplementary References [file ncomms9130-s1.pdf]

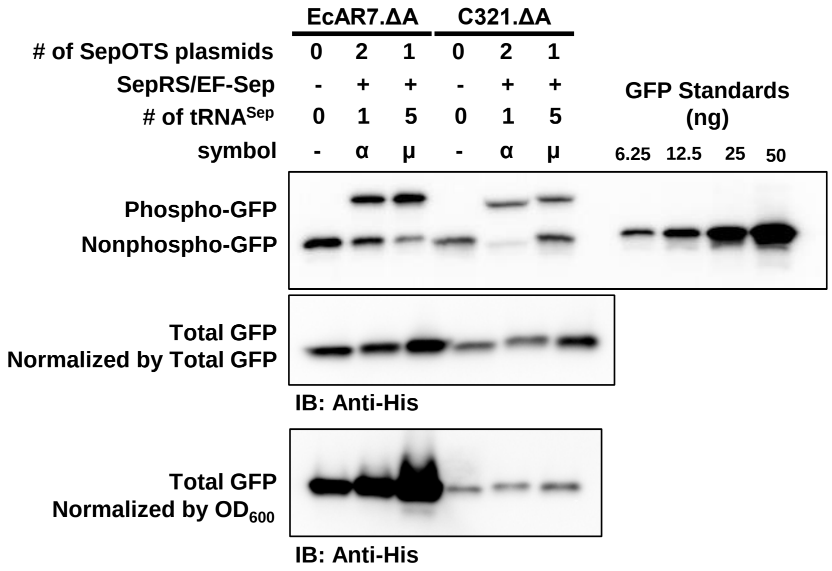

Supplementary Figure 1: **Representative blots from biological triplicate showing phospho-GFP expression at position E17TAG using two SepOTS variants in the EcAR7.ΔA versus C321.ΔA strains.** The level of GFP expression was assessed by western blot analysis (Anti-His). Normalized Phos-tag gel loading was approximated by regular SDS-PAGE analysis of total GFP expression to facilitate quantitation of phosphoprotein purity. Phosphoprotein purity was determined by Phos-tag gel shift of the GFP-normalized samples by comparing intensities of the upper band (phospho-GFP) and the lower band (nonphospho-GFP) from the western blot. OD_{600}-normalized expression levels shown in bottom blot.

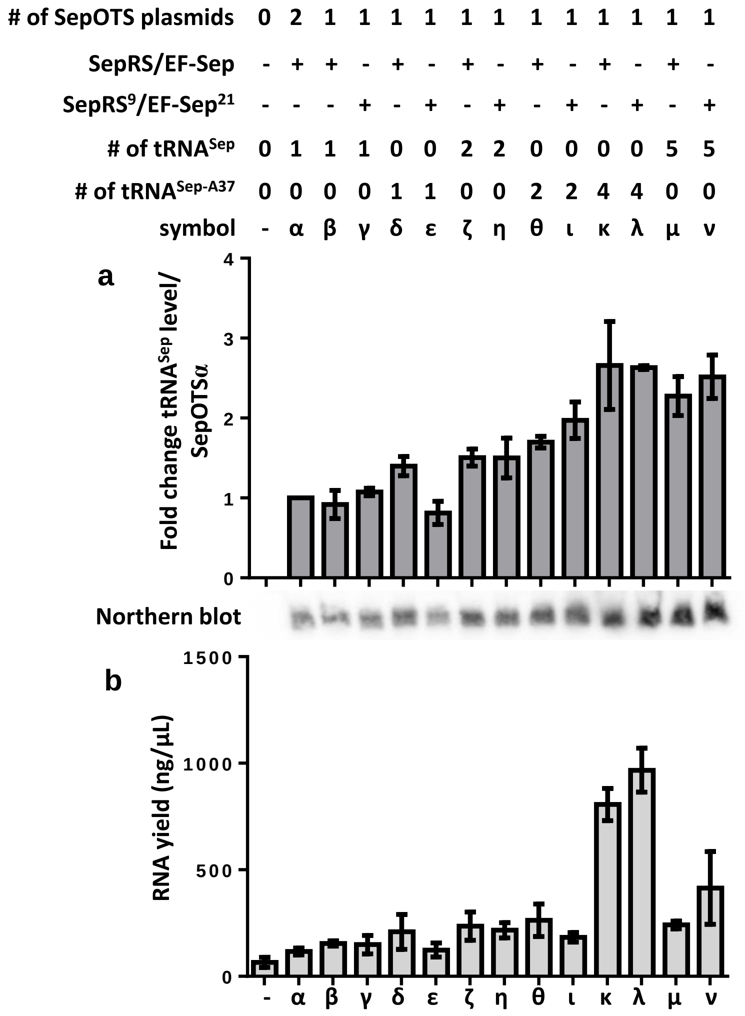

Supplementary Figure 2: **Determination of tRNA^{Sep} levels for different SepOTS variants in C321.ΔA.** (a) Northern blot analysis of tRNA^{Sep} levels from C321.ΔA cells containing different SepOTS variants after 20 hr expression of E17TAG GFP. Aliquots of the same cells as shown in Fig. 1c were used for RNA extraction and analyzed by northern blot. RNA was loaded on a 7% acrylamide gel containing 8 M urea and hybridized with a radioactive probe specific to tRNA^{Sep}. The same amount of total RNA measured by A260 was loaded in each lane (between 300-600

ng, depending on the set). Fold change reflects relative radioactive signal for $tRNA^{Sep}$ from each SepOTS variant compared to SepOTSα. Error bars report s.d. of three biological replicates. (b) Total RNA yields from C321.ΔA cells (equivalent number of cells to 1 mL OD 2.5) containing different SepOTS variants after 20 hr expression of E17TAG GFP in C321.ΔA. After RNA extraction, yields were quantified by A260 measurements. Error bars report s.d. of three biological replicates.

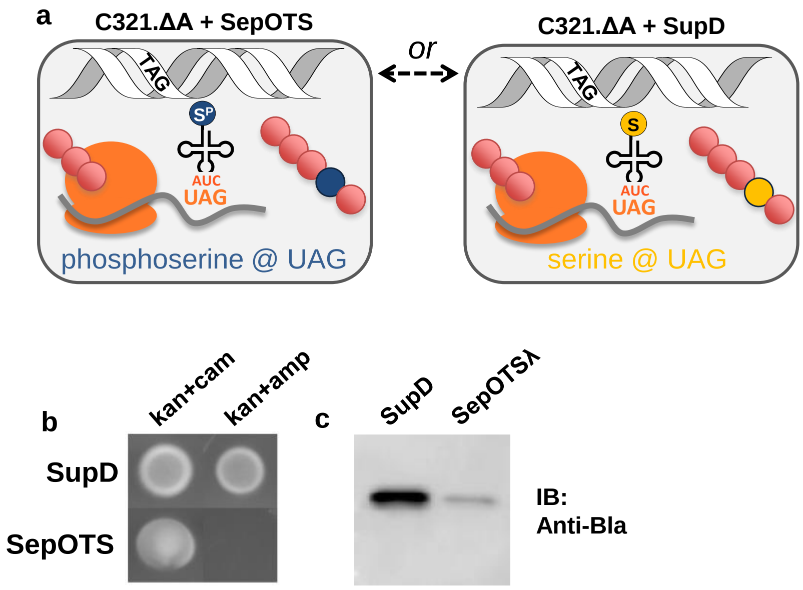

Supplementary Figure 3: **Amber codon permits incorporation of either serine or phosphoserine.** (a) Schematic of phosphorylated or non-phosphorylated protein production in C321.ΔA from a gene with the flexible UAG codon directing Sep insertion using SepOTS or Ser insertion using SupD. (b) Ampicillin resistance conferred by beta-lactamase rescue experiment. Kan^R plasmids containing SupD or SepOTSλ were tested with a dual-marker plasmid harboring a TAG-containing beta-lactamase gene (S36TAG) and a chloramphenicol acetyltransferase cassette. (c) Total beta-lactamase (S36TAG) expression confirmed by western blot.

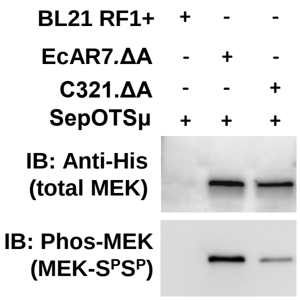

Supplementary Figure 4: **Comparison of MEK1-S^{P}S^P expression in BL21, EcAR7.ΔA, and C321.ΔA.** Western blot analysis of phosphorylated MBP-MEK1 (MBP-MEK1-S^{P}S^P) where TAG codons direct Sep to positions S218 and S222 in the MEK1 kinase activation loop. Expression of total and phospho-MBP-MEK1 is compared across BL21 RF1+, EcAR7.ΔA, and C321.ΔA strains using SepOTSμ. Loading was normalized by OD_{600} measurement.

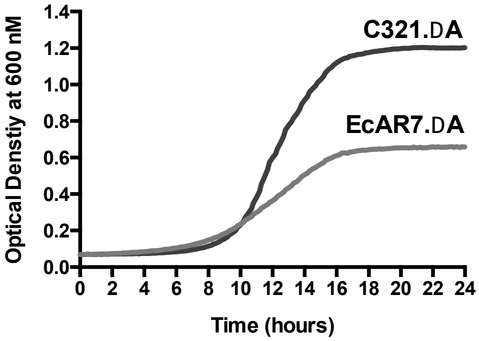

Supplementary Figure 5: **Growth curves for EcAR7.ΔA and C321.ΔA expressing MBP-MEK1 S218TAG/S222TAG with SepOTSμ.** Average values shown of 5 technical replicates for each strain.

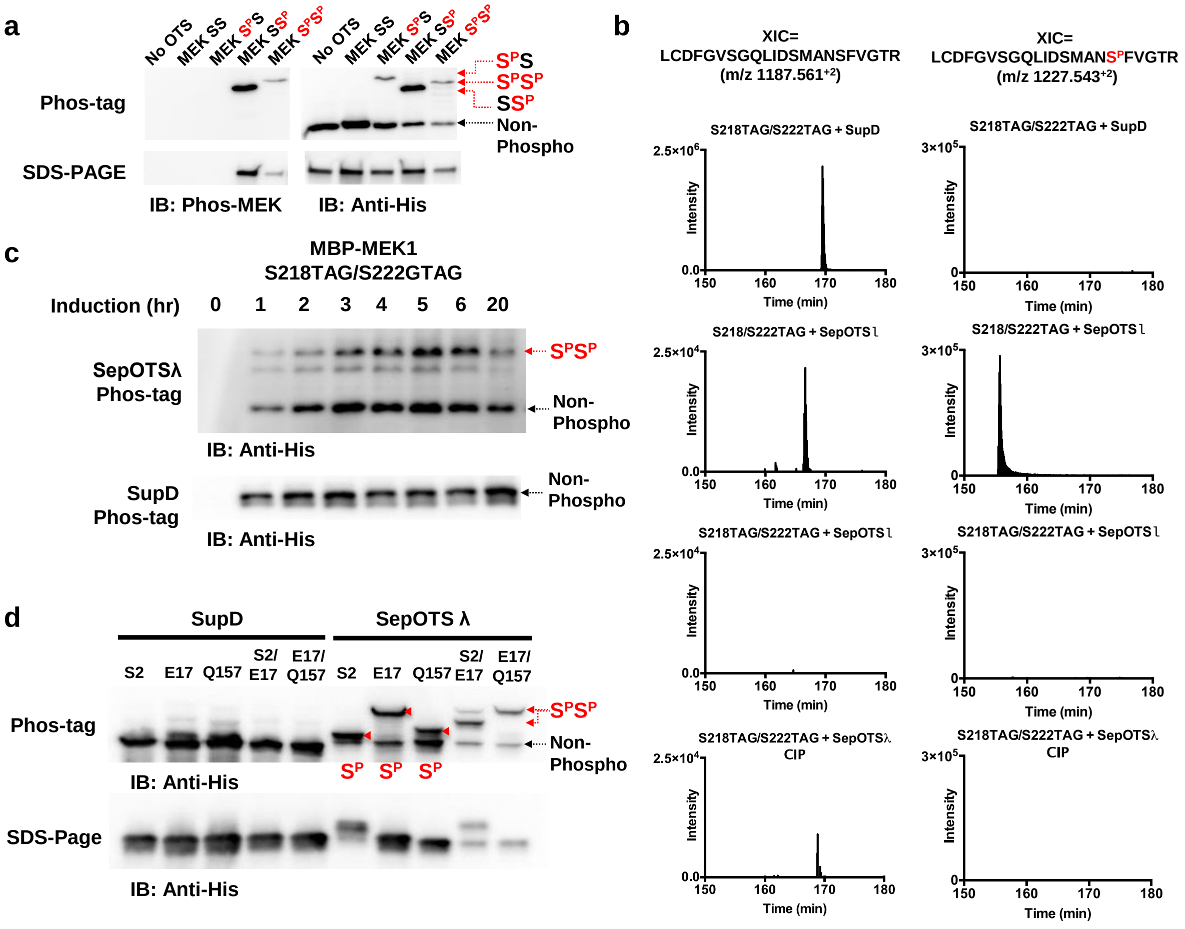

Supplementary Figure 6: **Analysis of positional phosphoserine incorporation bias.** (a) Expression of MBP-MEK1 variants was evaluated by western analysis using Anti-His antibodies. A phospho-specific antibody (Anti-Phos-MEK; Phospho-MEK1/2 (Ser217/221), 9154, Cell Signaling Technology) reported to identify MEK-S^PS^P was shown to react with both MEK-S^PS^P and MEK-SS^P. Phos-tag westerns showed distinct and reproducible shifts for each phosphorylated MEK variant. (b) MBP-MEK1 variants S218/S222TAG expressed using SepOTSλ and S218TAG/S222TAG expressed using SepOTSλ or SupD were evaluated by mass spectrometry. We could detect peptides with serine at positions 218/222, as well as singly phosphorylated peptides (Supplementary Table 1) however it was not possible to observe the doubly phosphorylated peptide presumably due to poor ionization. We therefore devised an

alternative strategy that involved treating the purified 218/222 phosphorylated MEK1 protein with alkaline phosphatase *in vitro* and then preparing tryptic digests for LC-MS/MS. We observed serine at positions 218 and 222 only in the phosphatase-treated protein and confirmed that the serine peptide is absent in untreated samples. Extracted ion chromatograms (XICs) are plotted for key peptides from the samples indicated. (c) Time course expression of MBP-MEK1 S218TAG/S222TAG in C321.ΔA with either SepOTSλ or SupD. Phos-tag gel shift shows that the highest levels of phospho-MBP-MEK1 are attained 5 hrs post-induction. (d) Demonstration of positional bias in Sep incorporation and/or stability using several singly and doubly phosphorylated forms of GFP expressed in C321.ΔA with either SupD or SepOTSλ. Samples analyzed after 5 hrs of expression by Phos-tag gel shift. Each construct had distinct and reproducible migration patterns.

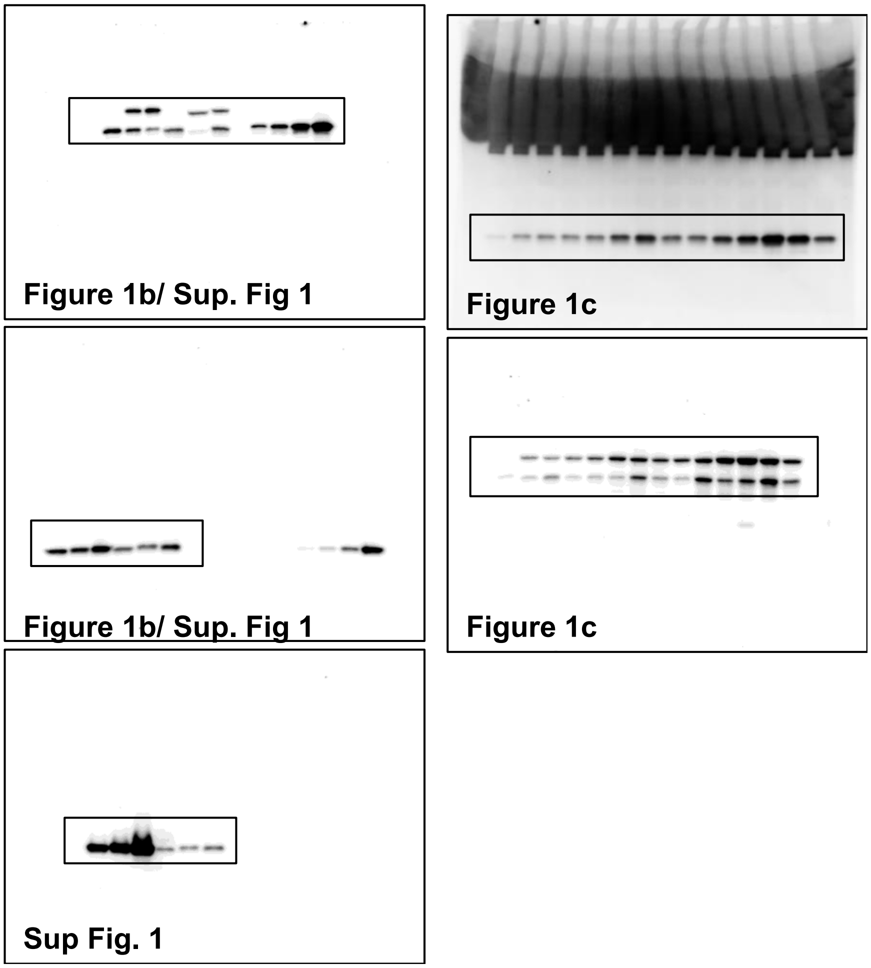

Supplementary Figure 7: **Full scans for western blots displayed in Figure 1 or Supplementary Figure 1**

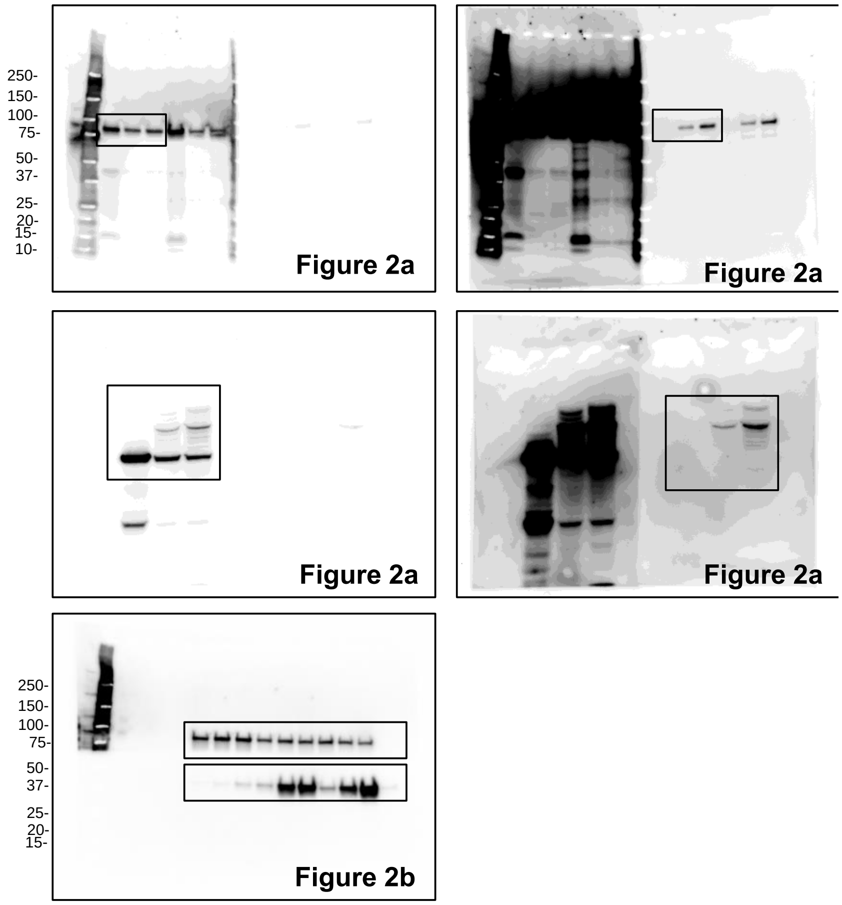

Supplementary Figure 8: **Full scans for western blots displayed in Figure 2**

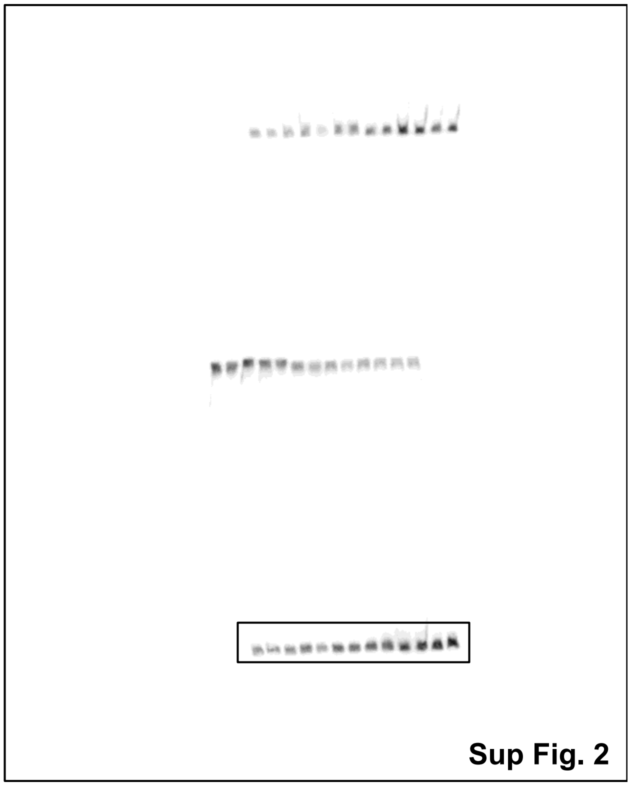

Supplementary Figure 9: **Full scan for Northern blot displayed in Supplementary Figure 2**

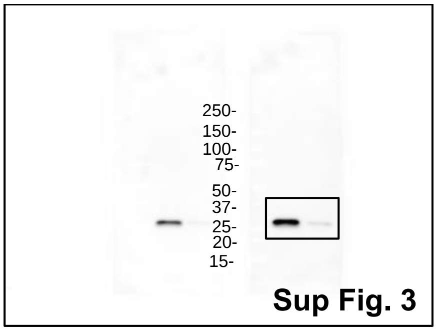

Supplementary Figure 10: Full scan for western blot displayed in Supplementary Figure 3

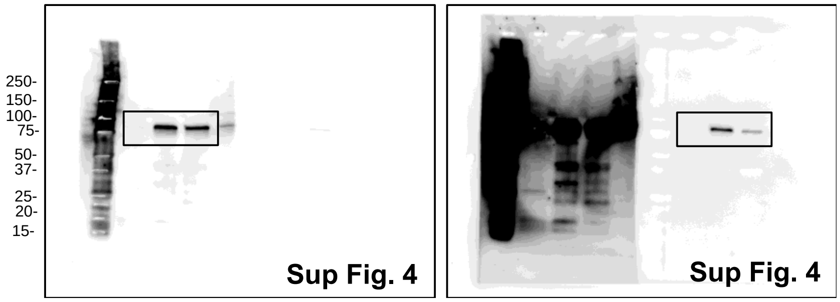

Supplementary Figure 11: Full scans for western blots displayed in Supplementary Figure 4

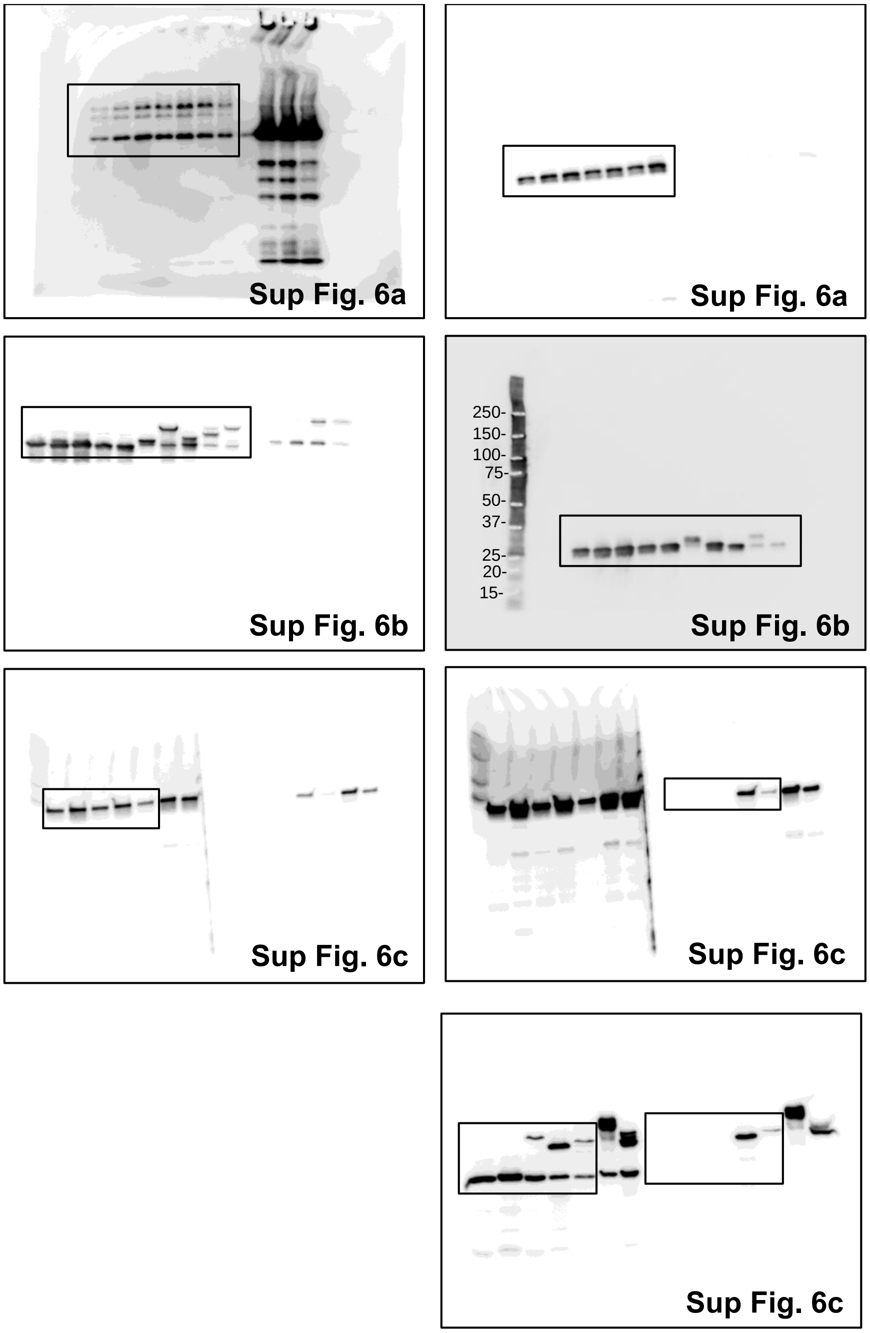

Supplementary Figure 12: **Full scans for western blots displayed in Supplementary Figure 6**

Modified sequence	Charge	m/z	Mass	Mass Error [ppm]	PEP	Score	218(TAG)/ 222(TAG)+ SupD	218(S)/ S222(TAG) + SepOTSλ	218(TAG)/ 222(TAG)+ SepOTSλ	218(TAG)/ 222(TAG) +SepOTSλ+ CIP
L**C**DFGVSGQLIDK	2	726.3636	1450.7126	-1.87	3.53E-04	56	(+)		(+)	(+)
MANSFVGTR	2	491.7424	981.4702	2.30	3.70E-02	34	(+)			(+)
L**C**DFGVSGQLIDSMANSFVGTR	3	792.0437	2373.1093	-0.92	3.98E-04	36	(+)			(+)
L**C**DFGVSGQLIDSMANSFVGTR	2	1187.5619	2373.1093	0.17	7.65E-35	134	(+)	(+)		(+)
L**C**DFGVSGQLIDSMANGFVGTR	3	782.0402	2343.0988	0.11	7.43E-14	77		(+)		(+)
L**C**DFGVSGQLIDSMANK	2	927.9399	1853.8652	-0.44	1.77E-12	111		(+)		
L**C**DFGVSGQLIDSMANQFVGTR	3	805.7192	2414.1359	0.16	7.15E-04	31		(+)		
L**C**DFGVSGQLIDSMANYFVGTR	2	1225.5776	2449.1406	-0.02	9.59E-09	60		(+)		
L**C**DFGVSGQLIDSM(ox)ANS(ph)FVGTR	3	824.0308	2469.0706	0.20	5.49E-06	48		(+)		
L**C**DFGVSGQLIDSMANS(ph)FVGTR	2	1227.5451	2453.0757	-0.09	2.18E-19	98		(+)		
L**C**DFGVSGQLIDGMANSFVGTR	3	782.0402	2343.0988	-0.44	1.91E-02	19			(+)	
L**C**DFGVSGQLIDQMANQFVGTR	2	1228.5885	2455.1624	-0.18	9.02E-09	61			(+)	
L**C**DFGVSGQLIDQMANYFVGTR	3	831.0630	2490.1672	1.66	1.78E-02	20			(+)	
L**C**DFGVSGQLIDR	2	740.3667	1478.7188	-0.98	9.00E-07	98			(+)	(+)
L**C**DFGVSGQLIDQMANSFVGTR	2	1208.0752	2414.1359	0.26	1.31E-05	45				(+)

Samples: MBP-MEK1 S218TAG/S222TAG with SupD or SepOTSλ or MBP-MEK1 S218/S222TAG + SepOTSλ; Fixed modification, Carbamidomethyl (**C**); Variable modification, Phosphorylation (ph); Oxidation (ox); Calf Intestinal Phosphatase (CIP)

Supplementary Table 1: Significant MBP-MEK1 peptides identified by mass spectrometry. Metrics shown for representative peptides. Peptides listed include only peptides unambiguously identified by MaxQuant (Andromeda search engine) or manually verified.

Primer ID	Sequence (5’ to 3’)
P1	GAGGAGAAAGGTACCATGTAGAAAGGAGAAGAACTTTTC
P2	GAAAAGTTCTTCTCCTTTCTACATGGTACCTTTCTCCTC
P3	CGACTAGATGGCTAACTAGTTCGTTGGCACCCGTTC
P4	GAACGGGTGCCAACGAACTAGTTAGCCATCTAGTCG
P5	GACTAGATGGCTAACTCTTTCGTTGGCACCCGTTC
P6	GAACGGGTGCCAACGAAAGAGTTAGCCATCTAGTC
P7	GGTCAGCTGATCGACTCTATGGCTAACTAGTTCG
P8	CGAACTAGTTAGCCATAGAGTCGATCAGCTGACC
P9	CAAAGTTCGAGTAGCTATCCGTAAAATCAGCCCCTTTGAG
P10	CTCAAAGGGGCTGATTTTACGGATAGCTACTCGAACTTTG
P11	ACCGCGGCCGCAAAAAAAATCCTTAGCTTTCG
P12	AAAGCGGCCGCGCTTCTTTGAGCGAAC
P13	CACGTAAGGCGGATTTAGAGTCCGCTGCC
P14	GGCAGCGGACTCTAAATCCGCCTTACGTG
P15	CTTGGTTGAGTACTCACCAGTCACAG
P16	GTTTGCGCAACGTTGTTGCCATTGCTACAGG

Supplementary Table 2: Primer sequences

Gene ID	Sequence
G1 (2x tRNA$^{Sep\text{-}A37}$)	GCGGCCGCAAAAAAAATCCTTAGCTTTCGCTAAGGATCTGC<br>AGGTCGACTCTAGAGGATCCTGGAGCCGGGGGTGGGATTTG<br>AACCCACGTAAGGCGGATTTAGAGTCCGCTGCCTAACCCCT<br>AGACTACCCCGGCGAATTCAGCGTTACAAGTATTACACAAA<br>GTTTTTTATGTTGAGAATATTTTTTTGATGGGGCGCCACTTAT<br>TTTTGATCGTTCGCTCAAAGAAGCGCCGCGGCAAAAAAAAT<br>CCTTAGCTTTCGCTAAGGATCTGCAGGTCGACTCTAGAGGAT<br>CCTGGAGCCGGGGGTGGGATTTGAACCCACGTAAGGCGGATT<br>TAGAGTCCGCTGCCTAACCCCTAGACTACCCCGGCGAATTCA<br>GCGTTACAAGTATTACACAAAGTTTTTTATGTTGAGAATATT<br>TTTTTGATGGGGCGCCACTTATTTTTGATCGTTCGCTCAAAG<br>AAGCGCGGCCGC
G2 (2x tRNA SupD)	GCGGCCGCAAAAAAAATCCTTAGCTTTCGCTAAGGATCTGCA<br>GGTCGACTCTAGAGGATCCTGGCGGAGAGAGGGGGATTTGA<br>ACCCCCGGTAGAGTTGCCCCTACTCCGGTTTTAGAGACCGGT<br>CCGTTCAGCCGCTCCGGCATCTCTCCGAATTCAGCGTTACAA<br>GTATTACACAAAGTTTTTTATGTTGAGAATATTTTTTTGATGG<br>GGCGCCACTTATTTTTGATCGTTCGCTCAAAGAAGCGCCGCG<br>GCAAAAAAAATCCTTAGCTTTCGCTAAGGATCTGCAGGTCGA<br>CTCTAGAGGATCCTGGCGGAGAGAGGGGGATTTGAACCCCC<br>GGTAGAGTTGCCCCTACTCCGGTTTTAGAGACCGGTCCGTTC<br>AGCCGCTCCGGCATCTCTCCGAATTCAGCGTTACAAGTATTA<br>CACAAAGTTTTTTATGTTGAGAATATTTTTTTGATGGGGCGC<br>CACTTATTTTTGATCGTTCGCTCAAAGAAGCGCGGCCGC

Supplementary Table 3: Synthetic gene sequences

Supplementary Methods

Strain and plasmid construction

BL21 RF1+ and EcAR7.ΔA strains were previously described[1,2]. C321.ΔA was derived from a previously described strain[3] with the following genotype modifications: Δ*mutS:zeo*, Δ*tolC*, Δ*bla:tolC*, *SerB*-/Δ*SerB*. SerB is a phosphoserine phosphatase, and deletion is required for sufficient levels of Sep in the cytoplasm for protein synthesis.[1]

The C-terminally His-tagged E17TAG GFP, Q157TAG and E17TAG/Q157TAG were each subcloned using the KpnI/HindIII sites from the modified pCR®-Blunt II-TOPO (Invitrogen) plasmid[2] into a modified pCRT7 NT Topo tetR pLtetO plasmid. The S2TAG and S2TAG/E17TAG substitutions were introduced by site-directed mutagenesis with primers P1 & P2 using the WT GFP and E17TAG GFP templates in pCRT7 NT Topo tetR pLtetO plasmid (all primer sequences are listed in **Supplementary Table 2**). The gene for the MEK1 fusion construct with N-terminal maltose binding protein (MBP-MEK1) was subcloned from the previously reported pCG-MBPMEK1XE plasmid[1] (X= S218TAG) using XbaI/HindIII restrictions sites. The fragment containing MBP-MEK1 was blunted using quick blunting enzyme (New England Biolabs, NEB) and treated with calf intestinal alkaline phosphatase (CIP). The fragment between the KpnI/HindIII sites in pCRT7 tetR pLtetO was removed and the restriction sites were blunted. The MBP-MEK1 fragment was ligated into the blunted KpnI/HindIII sites of the pCRT7 NT Topo tetR pLtetO plasmid. Ser222 was then mutated to TAG by site-directed mutagenesis using primers P3 & P4 to create MBP-MEK1 S218TAG/S222TAG. MBP-MEK1 S218TAG/S222 and S218/S222TAG were created from MBP-MEK1 S218TAG/S222TAG using site-directed mutagenesis with primers P5 & P6 and P7 & P8, respectively.

The previously described pET15-ERK2 plasmid[1] was used to construct a K54R mutation in ERK2 which inactivates the kinase. Lys54 was mutated by site-directed mutagenesis using primers P9 & P10.

The two SepOTSα plasmids were previously described as pSepT and pKD-SepRS-EFSep plasmids[1].

SepOTSβ, SepOTSζ, and SepOTSμ were constructed by combining the SepRS, EFSep, and $tRNA^{Sep}$ components of the pSepT and pKD-SepRS-EFSep plasmids[1] to create a one-plasmid OTS[4]. The 250 bp $tRNA^{Sep}$ cassette was PCR-amplified from the pSepT plasmid using primers P11 & P12. The PCR primers added NotI restriction sites to each end of the PCR product. The pKD-SepRS-EFSep plasmid was linearly digested with NotI and one (SepOTSβ), two (SepOTSζ) or five (SepOTSμ) copies of the $tRNA^{Sep}$ cassette were ligated sequentially. We obtained a modified version of the previously described pKD-SepRS9-EFSep21 plasmid as a generous gift from Dr. Dieter Söll[5]. It had been further modified to include one copy of the $tRNA^{Sep}$ in the same fashion using NotI as described above (SepOTSγ). To incorporate additional copies of $tRNA^{Sep}$, we digested the pKD-SepRS9-EFSep21 vector using MluI and AsiSI restriction sites and ligated in the two (SepOTSη) or five (SepOTSν) $tRNA^{Sep}$ copies generated using the restriction sites from plasmids described above. pKD-SepRS-EFSep plasmid containing 1x $tRNA^{Sep\text{-}A37}$ (SepOTSδ) was generated by mutating position G37 in $tRNA^{Sep}$ to A37 in the SepOTSβ plasmid using primers P13 & P14. To generate pKD-SepRS-EFSep plasmid containing 2x $tRNA^{Sep\text{-}A37}$ (SepOTSθ), we had the sequence G1 synthesized from Genewiz provided in the pUC57 vector (synthetic gene sequences shown in **Supplementary Table 3**). The 2x $tRNA^{Sep\text{-}A37}$ cassette was digested from the pUC57 vector using NotI restriction sites flanking both sides of the fragment. 1x $tRNA^{Sep}$ was removed from the SepOTSδ plasmid

using NotI and the 2x $tRNA^{Sep\text{-}A37}$ cassette was ligated into digested plasmid. The 4x $tRNA^{Sep\text{-}A37}$ pKD-SepRS-EFSep construct (SepOTSκ) was generated by adding an additional blunted 2x $tRNA^{Sep\text{-}A37}$ NotI fragment from pUC57 ligated into the blunted BglI restriction site in SepOTSθ. To insert the 1x $tRNA^{Sep\text{-}A37}$ cassette into the pKD-SepRS9-EFSep21 vector, 1x $tRNA^{Sep\text{-}A37}$ was isolated from SepOTSδ using the flanking NotI restriction sites, the 1x $tRNA^{Sep}$ was removed from SepOTSγ, and the 1x $tRNA^{Sep\text{-}A37}$ gene was ligated in the NotI restriction site of SepOTSγ (SepOTSε). 2x $tRNA^{Sep\text{-}A37}$ (SepOTSι) and 4x $tRNA^{Sep\text{-}A37}$ (SepOTSλ) plasmids with the pKD-SepRS9-EFSep21 backbone were generated by digesting SepOTSε using MluI and AsiSI restriction sites to remove the 1x $tRNA^{Sep\text{-}A37}$ cassette and inserting the 2x or 4x $tRNA^{Sep\text{-}A37}$ fragments via ligation. The 2x $tRNA^{SupD}$ sequence G2 was synthesized by Genewiz and provided in the Genewiz pCK vector.The 2x $tRNA^{SupD}$ was digested from pCK using flanking NotI restriction sites. NotI was used to remove the 1x $tRNA^{Sep}$ cassette from SepOTSβ, and the 2x $tRNA^{SupD}$ fragment was inserted into this position. To obtain the beta-lactamase S36TAG plasmid, a chloramphenicol acetyltransferase gene was inserted in the pCRT7 NT Topo tetR pLtetO vector between blunted XbaI sites. The beta-lactamase gene was mutated at position S36 to TAG via site-directed mutagenesis using primers P15 & P16.

Northern blot

Cell pellets corresponding to 1 mL of OD_{600} 2.5 of cells were resuspended in 0.5 mL of RNA extraction buffer (0.3 M sodium acetate, 10 mM EDTA, pH 4.5). 0.5 mL of acidic phenol was added to each tube, and samples were vortexed for 10 sec. Samples were then incubated on ice for 15 min, with quick vortexing every 3 min. Samples were then centrifuged for 12 min at 12,000 x g at 4 °C. The aqueous phase was transferred to a new tube, and 0.25 mL of RNA extraction buffer was added to each tube, vortexed, and centrifuged again. The aqueous phases

were combined. 700 μL of ethanol was added to each tube, and tubes were frozen at -80 °C overnight. Samples were then spun at 13,000 x g for 20 min at 4 °C and supernatant was removed. Pellets were washed with 1 mL ice cold 70% ethanol and centrifuged at 13,000 x g for 12 min at 4 °C. Ethanol was removed and pellets were allowed to dry. 30 μL of 200 mM Tris-HCl pH 8.0 were added to each sample and samples were incubated for 30 min at 37 °C for deacylation. RNA concentrations were determined by A260 measurement. Samples were run on a 7% acrylamide gel with 8 M urea. Sample loading was normalized by RNA concentration to the lowest concentrated sample, so the same total amount of RNA was loaded in each lane. This was repeated in biological triplicate using aliquots of the exact same samples analyzed for GFP expression level in Fig. 1c. Loading amounts between sets varied from approximately 300 to 600 ng per lane.

RNA was then transferred to a Hybond-XL membrane (Amersham) at 200 mA for 1 hr at 4 °C in 0.5x TBE in a Bio-Rad Trans-Blot cell, and then crosslinked using a UV Stratalinker™ 1800 (Stratagene). tRNA was hybridized using DNA probe with the sequence TGCCTAACCCCTAGACTACCCCGGC 5' labeled with radioactive [γ-^{32}P]ATP, which is specific for $tRNA^{Sep}$ bases 1-26 (identical in $tRNA^{Sep}$ and $tRNA^{Sep\text{-}A37}$)[6]. Images were collected using a Typhoon FLA9000 and densitometry was performed using ImageQuant TL v7.0 (GE Healthcare).

S36TAG beta-lactamase plate assays

C321.ΔA cells containing the pCRT7 NT Topo tetR pLtetO plasmid including beta-lactamase S36TAG and either SepOTSλ or the SupD plasmid were grown from glycerol stocks, induced, and grown for 20 hrs as for E17TAG GFP protein expression above. Cultures were diluted to OD_{600} of 2.0AU in LB media and 2 μL of cells were spotted on LB agar plates with

either kanamycin (25 μg per mL) and chloramphenicol (10 μg per mL) or kanamycin (25 μg per mL) and ampicillin (100 μg per mL). Plates were grown ~20 hrs at 30 °C and images were acquired with a Bio-Rad Gel Doc imager.

The same cultures used for the plate assays were run on a 4-15% acrylamide SDS-PAGE gel (1 μL lysate in 7.5 μL 1x Laemmli buffer per lane) and then transferred to PVDF. Beta-lactamase western blots were carried out with 1:1000 anti-β-lactamase antibody (Beta-lactamase SC-66062, Santa Cruz, Inc) followed by 1:10,000 DAM-HRP (Peroxidase-conjugated AffiniPure Donkey Anti-Mouse IgG, 715-035-150, Jackson ImmunoResearch). Signal was detected by enhanced chemiluminescence (Bio-Rad) imaged on a ChemiDoc™ XRS+CCD camera.

Growth curves

EcAR7.ΔA and C321.ΔA strains containing the MBP-MEK1 (S218TAG/S222TAG) and SepOTSμ plasmids were freshly streaked from glycerol stocks on LB agar plates supplemented with ampicillin (100 μg per mL), kanamycin (25 μg per mL) and 0.08% glucose. 5 mL of selective LB media supplemented with 0.08% glucose was inoculated with 5 colonies and grown to confluency. The precultures were back diluted to OD_{600} of 0.8 AU then diluted 1:100 into a 96-well cell culture plate (Costar 3595, Corning) containing LB media with 0.08% glucose, antibiotics, 2 mM Sep, 100 ng per mL ATC, and 1 mM IPTG in 5x replicates for each culture. The plate was incubated and continuously shaken (medium speed) at 30 °C in a Biotek Synergy HT plate reader monitoring OD_{600} in 10 min intervals for 24 hrs.

MBP-MEK1 treatment with calf intestinal alkaline phosphatase (CIP)

Affinity-purified MBP-MEK1 S^PS^P protein (45 μg total protein) was treated with calf intestinal alkaline phosphatase (CIP, NEB) using 45 units of enzyme with 1x NEB buffer 3 in a total reaction volume of 50 μL. The dephosphorylation reaction was carried out at 37 °C for 1 hr.

Samples were then dialyzed twice against 1 L cold 10 mM Tris-HCl buffer pH 8.0 using Slide-A-Lyzer^{TM} MINI devices with 2 kDa molecular weight cutoff (Life Technologies).

Protein digestion and mass spectrometry

Mass spectrometry of MBP-MEK1 (S218TAG/S222TAG expressed with SupD or SepOTSλ or S218/S222TAG expressed with SepOTSλ) was performed to confirm the incorporation of Sep at the target sites. Affinity-purified buffer-exchanged protein in 10 mM Tris-HCl buffer pH 8.0 was used for all experiments. The protein concentration was determined by A280 spectroscopy on a NanoDrop 2000c spectrophotometer (Thermo Scientific). Aliquots corresponding to 5 µg total protein were frozen at -80 °C and then dried in a rotary vacuum centrifuge operated at room temperature and 0.3 mbar. Dried proteins were dissolved in 12.5 µl solubilization buffer consisting of 1 mM EDTA, 10 mM DTT, 0.5% acid labile surfactant II (ALS, Protea Biosciences) in 10 mM Tris-HCl pH 8.0 and the reaction was incubated for 35 min at 35 °C in a water bath. All buffers for mass spectrometry were prepared at 23 °C. Reactions were quenched for 30 sec on ice and then supplemented with 2 µl 1 M Tris-HCl buffer pH 8.0 and 4.67 µl freshly prepared 100 mM aqueous iodoacetamide (Sigma Aldrich, St. Louis, MO) solution. Alkylation of cysteines was performed for 30 min at room temperature and in the dark. The reaction was then quenched with 0.7 µl 200 mM DTT. Protein digestion with Lysyl Endopeptidase (Wako Chemicals) was performed for 4 hr at 37 °C using 1 µl (0.0007 AU) of the enzyme. The digest was then diluted with 113.3 µl water, 0.667 µl 1 M $CaCl_2$ and 13.33 µl 1 M Tris-HCl buffer pH 8.0. Finally, 1.8 µl of a 0.5 µg per µl sequencing grade porcine trypsin (Promega) was added and proteins were digested for 14-16 hrs at 37 °C. This resulted in a trypsin concentration of 6 ng per µl in the final digest. The digest was acidified with 12.5 µl 20% TFA solution and cleavage of the acid cleavable detergent proceeded for 15 min at room

temperature. Each digest was then passed serially through 2 C_{18} StageTips. StageTips were prepared with 2 1.07 mm diameter punches of C_{18} (# 2215) Empore disks (3M Company, St. Paul, MN) stacked into a 200 µl pipette tip. The StageTip preparation and desalting was essentially performed as described by Rappsilber[7] but using 80% acetonitrile and 0.1% TFA as an elution solvent. Eluted peptides were dried at room temperature in a vacuum centrifuge, reconstituted in 10 µl 50% acetonitrile with 0.1% formic acid and then quantified by A280. An aliquot corresponding to 500 ng peptides was transferred into a glass HPLC vial, dried with a gentle stream of nitrogen. The dried peptides were reconstituted in 10 µl LC-MS sample buffer consisting of 5% DMSO, 5 mM sodium phosphate prepared in a solvent mixture of 3:8 by volume of 70% formic acid and 0.1% TFA. A total of 3 µl of this solution corresponding to 150 ng peptides were injected for LC-MS/MS analysis.

LC-MS/MS experiments were performed on an Orbitrap Velos instrument operated with a top 10 higher energy collisional dissociation (HCD) method according to Lajoie[3]. Peptides were separated by nanoflow liquid chromatography on a nanoACQUITY UPLC System (Waters) essentially as described[3] with the following minor modifications. The trap column consisted of a 360 µm OD and 150 µm ID fused silica capillary terminated with a 2-3 mm long Kasil frit. The frit was prepared according to the instructions of a commercially available frit kit (Next Advance). The trap column was slurry packed with 3 µm diameter Reprosil-Pur 120 C18-AQ C_{18} particles (Dr. Maisch GmBH) to a length of 30 mm using methanol as the packing solvent. The analytical column consisted of a 75 µm ID PicoFrit column (New Objectives) slurry packed to a length of 20 cm with 1.9 µm diameter particles Reprosil-Pur 120 C18-AQ C_{18} particles (Dr. Maisch GmBH). Methanol was used as the packing solvent. Eluent A and Eluent B were 0.1% formic acid and 0.1% formic acid in acetonitrile respectively. The injection volume was 3 µl.

Peptides were trapped for 6 min with a flow rate of 2.5 µl per min with 2% eluent B. Gradient elution of peptides was performed at a flow rate of 300 nl per min with the following 200 min gradient program: (min/%B) 0.0/2.0, 2.0/7.0, 166.0/30.0, 176.0/55.0, 177.0/95.0, 182.0/95.0, 183.0/2.0, 200.0/2.0.

Bioinformatics

Spectra from shotgun discovery experiments were matched with MaxQuant[8] version 1.5.1.2 using the default search parameters considering the variable modifications oxidation (M) deamidation (N/Q) and the fixed modification carbamidomethyl(C). The enzyme specificity was Trypsin/P. Only fully tryptic peptides with up to 3 missed cleavages were considered. The precursor mass tolerance was 4.5 ppm and the fragment ion mass tolerance was 20 ppm and the false discovery rate for protein and peptide identifications was 1%. Identified peptides were reported after removing reverse database hits in Microsoft Excel. A recently described method for detection of natural amino acids and non-standard amino acids in proteins[4] was adapted for the MBP-MEK1 protein. Briefly, spectra were matched against the *E. coli* protein database EcoCyc[9] v.17 and a custom database representing 64 MEK protein sequences. The MEK database contained the 8 amino acids S, Q, K, Y, G, T, V and R, which have been previously shown to be incorporated at amber codons using the Sep OTS system[4]. The MEK protein sequence used in this work is provided below. The position of amber codons is marked by **X.**

MKIEEGKLVIWINGDKGYNGLAEVGKKFEKDTGIKVTVEHPDKLEEKFPQVAATGDGPDIIFWA
HDRFGGYAQSGLLAEITPDKAFQDKLYPFTWDAVRYNGKLIAYPIAVEALSLIYNKDLLPNPPKT
WEEIPALDKELKAKGKSALMFNLQEPYFTWPLIAADGGYAFKYENGKYDIKDVGVDNAGAKA
GLTFLVDLIKNKHMNADTDYSIAEAAFNKGETAMTINGPWAWSNIDTSKVNYGVTVLPTFKGQP
SKPFVGVLSAGINAASPNKELAKEFLENYLLTDEGLEAVNKDKPLGAVALKSYEEELAKDPRIAA
TMENAQKGEIMPNIPQMSAFWYAVRTAVINAASGRQTVDEALKDAQTNSSSNNNNNNNNNNL
GIEGRENLYFQGHMPKKKPTPIQLNPAPDGSAVNGTSSAETNLEALQKKLEELELDEQQRKRLEA
FLTQKQKVGELKDDDFEKISELGAGNGGVVFKVSHKPSGLVMARKLIHLEIKPAIRNQIIRELQVL
HECNSPYIVGFYGAFYSDGEISICMEHMDGGSLDQVLKKAGRIPEQILGKVSIAVIKGLTYLREKH
KIMHRDVKPSNILVNSRGEIKLCDFGVSGQLID**X**MAN**X**FVGTRSYMSPERLQGTHYSVQSDIWS
MGLSLVEMAVGRYPIPPPDAKELELMFGCQVEGDAAETPPRPRTPGRPLSSYGMDSRPPMAIFEL
LDYIVNEPPPKLPSGVFSLEFQDFVNKCLIKNPAERADLKQLMVHAFIKRSDAEEVDFAGWLCSTI
GLNQPSTPTHAAGVAAAAAHHHHHH*

Peptides matching to the sequence of the tryptic reporter peptide LCDFGVSGQLIDXMANXFVGTR are reported in Supplementary Table 1.

Extracted ion chromatograms of selected reporter peptides were extracted from raw LC-MS/MS data using Xcalibur software v. 3.0.69 (Thermo Scientific) with a mass tolerance of 10 ppm and plotted using Prism (GraphPad).

Supplementary References:

1. Park, H.S. et al. Expanding the genetic code of *Escherichia coli* with phosphoserine. *Science* **333**, 1151-1154 (2011).
2. Heinemann, I.U. et al. Enhanced phosphoserine insertion during Escherichia coli protein synthesis via partial UAG codon reassignment and release factor 1 deletion. *FEBS Lett* **586**, 3716-3722 (2012).
3. Lajoie, M.J. et al. Genomically recoded organisms expand biological functions. *Science* **342**, 357-360 (2013).
4. Aerni, H.R., Shifman, M.A., Rogulina, S., O'Donoghue, P. & Rinehart, J. Revealing the amino acid composition of proteins within an expanded genetic code. *Nucleic Acids Res* doi:gku1087 (2014).
5. Lee, S. et al. A facile strategy for selective incorporation of phosphoserine into histones. *Angew. Chemie Int. Ed. Engl* **52**, 5771-5775 (2013).
6. Sauerwald, A. et al. RNA-dependent cysteine biosynthesis in archaea. *Science* **307**, 1969-1972 (2005).
7. Rappsilber, J., Mann, M. & Ishihama, Y. Protocol for micro-purification, enrichment, pre-fractionation and storage of peptides for proteomics using StageTips. *Nat. Protocols* **2**, 1896-1906 (2007).
8. Cox, J. & Mann, M. MaxQuant enables high peptide identification rates, individualized p.p.b.-range mass accuracies and proteome-wide protein quantification. *Nat. Biotechnol.* **26**, 1367-1372 (2008).
9. Keseler, I.M. et al. EcoCyc: fusing model organism databases with systems biology. *Nucleic Acids Res* **41**, D605-612 (2013).
